# Seasonal Dynamics of Eukaryotic Microbial Communities in the Water-Receiving Reservoir of the Long-Distance Water Diversion Project, China

**DOI:** 10.3390/microorganisms12091873

**Published:** 2024-09-11

**Authors:** Yingying Yang, Fangfang Ci, Ailing Xu, Xijian Zhang, Ning Ding, Nianxin Wan, Yuanyuan Lv, Zhiwen Song

**Affiliations:** 1 School of Environmental and Municipal Engineering, Qingdao University of Technology, Qingdao 266520, China; yangyingyingqd@163.com (Y.Y.); xalcsu@sina.com (A.X.);; 2 Qingdao Branch of Shandong Water Transfer Project Operation and Maintenance Center, Qingdao 266525, China; 3 Binzhou Branch of Shandong Water Transfer Project Operation and Maintenance Center, Binzhou 256600, China

**Keywords:** eukaryotic microorganisms, storage reservoir, seasonal dynamics, environmental driving factors, co-occurrence network patterns

## Abstract

Inter-basin water transfer projects, such as the Yellow River to Qingdao Water Diversion Project (YQWD), are essential for addressing water scarcity, but impact local aquatic ecosystems. This study investigates the seasonal characteristics of eukaryotic microbial communities in the Jihongtan Reservoir, the main water-receiving body of YQWD, over a one-year period using 18S rDNA amplicon sequencing. The results showed that the eukaryotic microbial diversity did not exhibit significant seasonal variation (*p* > 0.05), but there was a notable variance in the community structure (*p* < 0.05). Arthropoda and *Paracyclopina*, representing the most dominant phylum and the most dominant genus, respectively, both exhibited the lowest abundance during the winter. The Chlorophyta, as the second-dominant phylum, demonstrates its higher abundance in the spring and winter. The Mantel test and PLS-PM (Partial Least Squares Path Modeling) revealed that water temperature (WT), dissolved oxygen (DO), and pH influenced the seasonal dynamic of eukaryotic microbial communities significantly, of which WT was the primary driving factor. In addition to environmental factors, water diversion is likely to be an important influencing factor. The results of the co-occurrence network and robustness suggested that the spring network is the most complex and exhibits the highest stability. Moreover, keystone taxa within networks have been identified, revealing that these key groups encompass both abundant and rare species, with specificity to different seasons. These insights are vital for understanding the seasonal variation of microbial communities in the Jihongtan Reservoir during ongoing water diversions.

## 1. Introduction

Inter-basin water transfer projects represent a critical engineering intervention designed to address the discord between the escalating water demand and its availability [1]. The Yellow River to Qingdao Water Diversion Project (YQWD) was constructed to effectively mitigate the water scarcity in Qingdao, China. The Jihongtan Reservoir, serving as the only water-receiving reservoir of YQWD, also holds the distinction of being the largest man-made dammed plain reservoir in Asia. Since its completion in 1989, the reservoir has been in operation for 35 years, having transferred over 5 billion cubic meters of water from both the Yellow River and Yangtze River, making it responsible for more than 90% of the water used by the urban population of Qingdao [2]. Although the water diversion project has alleviated water shortages to a certain extent, it simultaneously changes the hydrological regime and the characteristics of receiving area water bodies and provides a path for biological invasions, subsequently affecting the local ecology [3,4]. 

Eukaryotic microbes are widely distributed in aquatic environments, serving as both producers of organic matter and major consumers of bacterial biomass, playing a significant ecological role in maintaining multifunctionality, facilitating material cycling, and contributing to energy transfers within food webs [5]. They have more complexly structured cellulars than prokaryotes, containing organelles with diverse functions, such as vacuolar constrictors for osmoregulation, peduncles for phagocytic feeding, and chloroplasts for photosynthesis. These structures may exhibit a wide range of responses to environmental heterogeneity, making eukaryotic microbes highly sensitive to local stresses [6,7,8]. Water reservoirs are highly dynamic environments that are influenced by local human activities, such as artificial water transfers, as well as regional climate changes like temperature and precipitation [9,10]. Therefore, driven by environmental heterogeneity, microorganisms in reservoirs are temporally variable. For example, it has been reported that the succession of eukaryotic microbial communities occurs along with the season [11,12,13]. Understanding the dynamic variations remained a considerable challenge due to the complexity of eukaryotic microbial communities, especially in water-receiving areas with obvious seasonality, facing environments under compounded long-term impacts [14]. It is crucial to monitor the dynamic changes in eukaryotic microorganisms in drinking water to provide early warning of microbial hazards and ensure the safe management of drinking water [15]. 

Both environmental factors and biological interactions can drive the dynamics of microorganisms in aquatic ecosystems [16]. Some studies have demonstrated that the community structure of eukaryotic microorganisms is associated with changes in environmental factors. Zhang et al. [17] reported that nutrients and temperature significantly influenced the eukaryotic microbial diversity in a drinking water reservoir in northwest China. Yang et al. [18]. investigated the relationship between eukaryotic microbial communities and environmental factors in extreme environments on the Tibetan Plateau, finding that the water temperature, pH, and total phosphorus were the primary driving factors. Co-occurrence network analysis is an effective method that has been extensively applied to explore the interconnectivity among microorganisms and elucidate the complexity and stability of these ecological associations based on topological characteristics [19,20]. A positive correlation can be interpreted as cooperative behaviors such as syntrophic interactions or shared environmental requirements. By contrast, negative relationships may be generally explained as competition as well as distinctive environmental niches and spatial isolation [21,22,23]. Beyond co-occurrence patterns, microbial networks can also be utilized to statistically recognize keystone species [24]. Microbial keystone taxa are highly connected taxa that individually or in a guild exert a considerable influence on the microbiome structure and functioning, having a unique and crucial role in microbial communities [25]. Recent studies have revealed that key taxa exert a positive influence on the stability of microbial symbiotic networks [26,27].

This study intends to investigate the seasonal characteristics of eukaryotic microorganisms in the Jihongtan reservoir under conditions of year-round water diversion, based on 18S rDNA amplicon sequencing technology. The specific objectives were (1) to investigate the seasonal variations in the community composition and diversity of eukaryotic microbial communities in the reservoir; (2) to identify environmental factors driving eukaryotic microbial diversity and explore how dominant environmental variables affect that diversity; and (3) to reveal the co-occurrence patterns of eukaryotic microbial communities in different seasons.

## 2. Materials and Methods

### 2.1. Study Area and Sample Collection

**Figure 1:**
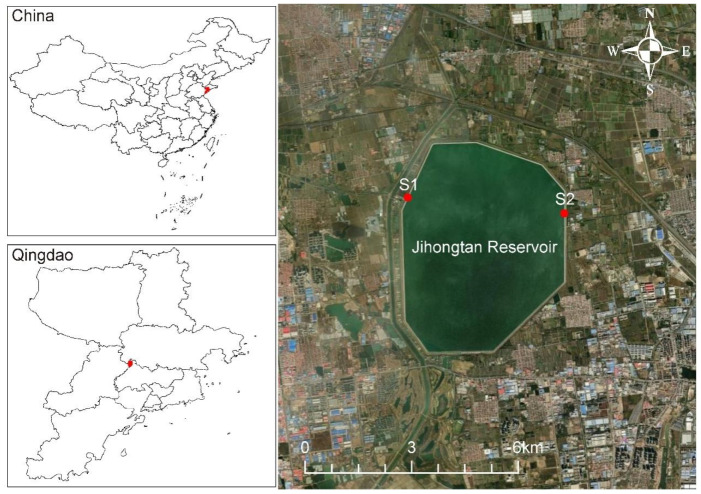
Geographical location of Jihongtan Reservoir and sampling sites.

Jihongtan Reservoir, located in Qingdao (36°20′55″~36°23′31″ N, 120°12′27″~120°14′55″ E), Shandong province, China, is an eight-sided storage reservoir for drinking water (Figure 1). The reservoir has a volume of approx. 1.46 × 10^8^ m^3^, with a design water level of 14.2 m, a dam length of 14.2 km, and a surface area of 14.4 km^2^. Water is transferred from the Eastern Route of the South-to-North Water Diversion Project (ESNWD), the Yellow River to Qingdao Water Diversion Project (YQWD), and Xiashan Reservoir (XR) through the aqueduct to the reservoir. ESNWD extracts water from the lower section of the Yangtze River, specifically from Sanjiangying in the Yangzhou area. It supplies water to Tianjin and the Jiaodong Peninsula of Shandong Province by utilizing the existing river channels of the Beijing–Hangzhou Grand Canal, connecting a chain of large lakes, including the Hongze, Luoma, Nansi, and Dongping Lakes. YQWD is located in Shandong province and it channels the water from the Yellow River in Binzhou City down to Jihongtan Reservoir in Qingdao, involving 6 cities of Binzhou, Dongying, Weifang, Yantai, Weihai, and Qingdao, with a total length of 290 km. XR is a drinking water reservoir in Weifang of Shandong Province, used to temporarily replenish the Jihongtan Reservoir during water shortages in summer and autumn. Table 1 shows the cumulative inflow amount of water diversion projects in four seasons. The local climate delineates the year into four distinct seasons.

To explore the eukaryotic microorganisms in the Jihongtan Reservoir throughout the four seasons, we collected water samples from 2 sites every month, obtaining 6 samples for each season and a total of 24 samples for the entire year. Sampling was conducted from site S1 (120°12′28″ E; 36°20′15″ N) and site S2 (120°14′55″ E; 36°22′22″ N), respectively located at the inlets and outlets of the reservoir (Figure 1). Then, 10 L water samples were collected 0.5 m below the water surface. All samples were collected at approximately 10:00–12:00 and examined within 48 h. 

### 2.2. Physicochemical Parameter Monitoring

Water temperature (WT), pH, and dissolved oxygen (DO) were measured by HQ40d (HACH, USA). Other physical and chemical parameters, including the total nitrogen (TN), total phosphorus (TP), chemical oxygen demand (COD), ammonium nitrogen (NH_4_^+^-N), nitrite nitrogen (NO_2_^−^-N), nitrate nitrogen (NO_3_^−^-N), and chlorophyll-a (Chl-a), were determined according to the environmental monitoring method standards of Ministry of Ecology and Environment of the People’s Republic of China [28]. Chl-a was determined according to Li et al [29]. 

### 2.3. DNA Extraction and High-Throughput Sequencing

The 0.22 μm filter membranes were used to filter the water samples, then cut into fractions for DNA extraction on 24 samples using the E.Z.N.A.^®^ soil DNA Kit (Omega Bio-tek, Norcross, GA, USA). The quality and concentration of DNA were determined by 1.0% agarose gel electrophoresis and a NanoDrop2000 spectrophotometer (Thermo Scientific, Waltham, MA, USA) and kept at −80 °C prior to further use. The amplification of the V4 region of the 18S rRNA was conducted using the primer set TAReuk454FWD1 (5′-CCAGCASCYGCGGTAATTCC-3′) and TAReukREV3R (5′-ACTTTCGTTCTTGATYRA-3′) [30], with amplicon length of 405 bp. PCR amplification cycling conditions were as follows: initial denaturation at 95 °C for 3 min, followed by 35 cycles of denaturing at 95 °C for 30 s, annealing at 55 °C for 30 s, extension at 72 °C for 45 s, single extension at 72 °C for 10 min, and end at 10 °C. The PCR product was extracted from 2% agarose gel and purified using the PCR Clean-Up Kit (YuHua, Shanghai, China) according to manufacturer’s instructions and quantified using Qubit 4.0 (Thermo Fisher Scientific, Waltham, MA, USA). The sequencing was completed on the Illumina MiSeq platform (Illumina, San Diego, CA, USA) using a paired-end (2 × 250 bp) approach by Majorbio Bio-Pharm Technology Co., Ltd. (Shanghai, China).

### 2.4. Sequencing Data Processing

Raw FASTQ files were processed using an in-house Perl script for de-multiplexing, followed by quality filtering with fastp v0.19.6 [31]. Sequences were merged using FLASH v1.2.7 [32] under specific criteria: (i) truncation at sites with an average quality score below 20 within a 50 bp sliding window, discarding the truncated reads shorter than 50 bp and those with ambiguous characters; (ii) assembly of overlapping sequences longer than 10 bp with a maximum mismatch ratio of 0.2 in the overlap region, discarding non-assemblable reads; and (iii) sample identification based on barcode and primer matching, allowing for 2 nucleotide mismatches in primers. The resultant sequences were clustered into operational taxonomic units (OTUs) using UPARSE v7.1 [33] at a 97% similarity threshold, with the most abundant sequence per OTU selected as the representative. Each representative sequence was assigned to taxonomic categories against the 18S rRNA database (Silva v138) using the Ribosomal Database Project (RDP) classifier [34] at a confidence threshold of 0.7. To minimize the effects of sequencing depth on alpha and beta diversity measure, the samples were resampled randomly to the lowest number of retrieved sequences across all samples.

### 2.5. Statistical Analysis 

Sequencing data in this study were processed using QIIME 2. A one-way analysis of variance (ANOVA) with a post hoc Tukey’s Honestly Significant Difference (HSD) test was carried out to examine the seasonal variations of water physicochemical properties and alpha diversity indices. Before conducting the ANOVA, tests for homogeneity and normality were performed. If the data were found to be non-normal, the non-parametric Kruskal–Wallis test was used to assess significant differences. Statistical significance was accepted as *p* < 0.05. The Venn diagram constructed with shared and unique operational taxonomic units (OTUs) was used to depict the similarities and differences among communities. Heatmap of the top 50 genera were generated via the “ComplexHeatmap” package (v 2.20.0) in R [35]. Ordination analysis of eukaryotic microbial communities was carried out using non-metric multidimensional scaling (NMDS) based on the distance algorithm of Aitchison [36]. Analysis of similarity (ANOSIM) was performed to investigate the variability of eukaryotic microbial communities between groups [37]. To determine the characteristics and variations of eukaryotic microorganisms in different seasons, the Linear discriminant analysis effect size (LEfSe) was utilized via the R package “microbiomeMarker” (v 1.10.0) [38]. We implemented a Mantel test to assess the correlations between environmental factors and eukaryotic microbial communities based on Spearman rank correlation, and the analysis was performed using the “linkET” package (v 0.0.7.4) [28]. To further reveal the effects of environmental variables on microbial community structure, a Partial Least Squares Path Model (PLS-PM) was conducted by R package “plspm” (v 0.5.1) [39]. Co-occurrence network analysis was performed to investigate the interactions between microbial composition. The computation of network properties and the identification of keystone taxa were carried out using the R package “ggClusterNet” (v 0.1.0) [40]. The network was visualized using the Gephi v0.9.2.

## 3. Results

### 3.1. Seasonal Variations in Water Physicochemical Properties

**Figure 2:**
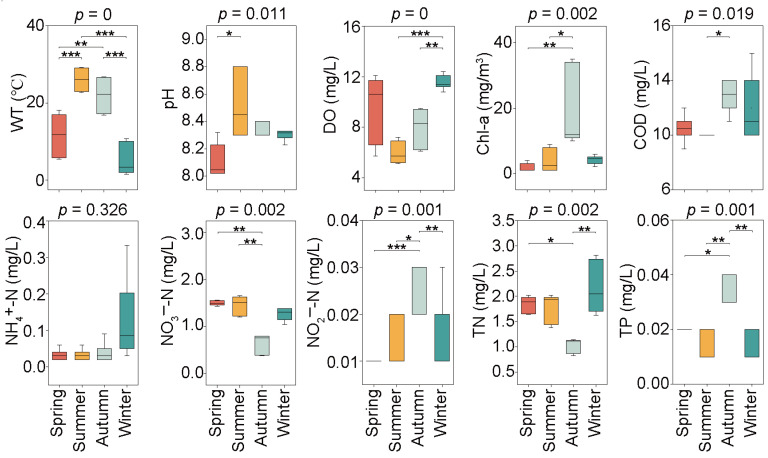
Seasonal variation of water physicochemical properties in Jihongtan Reservoir. (Significance: * *p* < 0.05, ** *p* < 0.01, *** *p* < 0.001). (WT: water temperature; DO: dissolved oxygen; Chl-a: chlorophyll-a; COD: chemical oxygen demand; NH_4_^+^-N: ammonia nitrogen; NO_3_^−^-N: nitrate nitrogen; NO_2_^−^-N: nitrite nitrogen; TN: total nitrogen; TP: total phosphorus).

The seasonal variations of water physicochemical properties, namely WT, pH, DO, COD, TN, TP, NH_4_^+^-N, NO_3_^−^-N, NO_2_^−^-N, and Chl-a, are shown in Figure 2. All the above-mentioned environmental variables, except for NH_4_^+^-N, exhibited significant seasonal differences (*p* < 0.05). A maximum average WT of 26.03 °C and minimum average WT of 5.17 °C were observed in the summer and winter, respectively. On the contrary, the highest average DO of 11.55 mg/L emerged in winter, and the lowest average DO of 5.97 mg/L was recorded in summer. pH mean values differed significantly between spring and summer, with a maximum value of 8.51 in the summer and a minimum value of 8.11 in the spring. The mean COD concentrations varied significantly between the summer and autumn, with the highest value of 12.8 mg/L in the autumn and the lowest value of 9.8 mg/L in the summer. TN, TP, NO_3_^−^-N, NO_2_^−^-N, and Chl-a did not show significant differences in the spring, summer, and winter, while the highest or lowest average concentration of these variables was exhibited in the autumn. Specifically, the minimum average NO_3_^−^-N and TN were observed in the autumn, with their values of 0.64 mg/L and 1.03 mg/L, respectively; the maximum average NO_2_^−^-N, TP, and Chl-a concentrations were detected in the autumn, with their values of 0.026 mg/L, 0.033 mg/L, and 19.00 mg/m^3^, respectively.

### 3.2. Seasonal Changes in OUTs and Alpha Diversity

**Figure 3:**
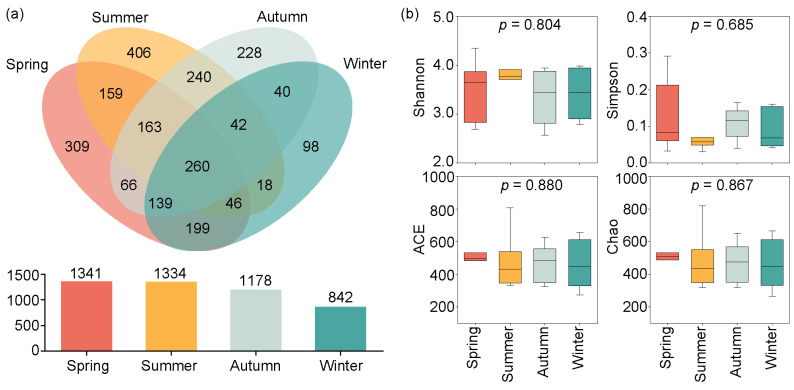
Seasonal variations in OTUs and Alpha diversity in Jihongtan Reservoir. (**a**) Venn diagram of the OTUs among the four seasons; (**b**) Alpha diversity indices of eukaryotic microbial communities in the four seasons.

The Venn diagram in Figure 3a indicates that 1341, 1334, 1178, and 842 OTUs were identified from spring to winter. Among these, 260 OTUs were shared across all seasons, while 309, 406, 228, and 98 OTUs were unique to the spring, summer, autumn, and winter, respectively. 

The Alpha diversity indices (Shannon, Simpson, ACE, and Chao indices) exhibited seasonal variations, but showed no significance (*p* > 0.05) (Figure 3b). The mean values of the four alpha diversity indices were higher in the spring and summer than in the autumn and winter. Specifically, the ACE and Chao indices showed the highest average values in the spring, but the lowest in the winter. The maximum mean values of the Shannon and Simpson indices occurred in the spring and summer, respectively; the minimum mean values of the Shannon and Simpson indices were observed in the autumn and winter, respectively. 

### 3.3. Eukaryotic Microbial Community Composition and Biomarkers Analysis

**Figure 4:**
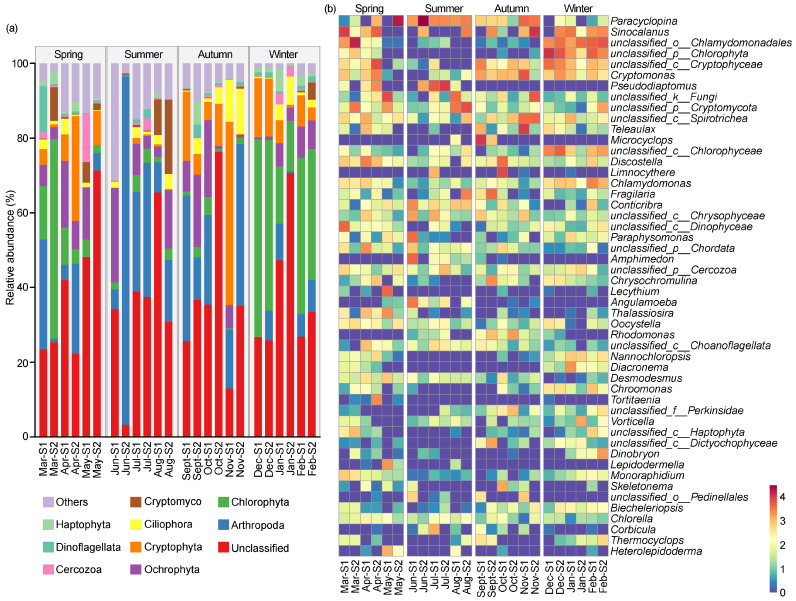
The compositions of eukaryotic microbial community on phyla level (**a**) and genus level (**b**) in Jihongtan Reservoir. Relative abundance less than 1% is defined as others.

Figure 4a reveals an overview of the eukaryotic microbial community composition of the top 1% phyla across different seasons. A total of 51 eukaryotic phyla were identified in the reservoir throughout the year, with the dominant phyla being Arthropoda (19.34%), Chlorophyta (13.36%), Ochrophyta (7.85%), Cryptophyta (7.64%), Ciliophora (3.19%), Cryptomycota (2.18%), Cercozoa (1.22%), Dinoflagellata (1.22%), and Haptophyta (1.21%). The relative abundances of the dominant taxa exhibited some seasonal dynamics. Arthropoda was the most dominant phylum in the spring (18.65%), summer (30.97%), and autumn (22.24%); however, its dominance decreased in winter, accounting for only 5.52%, making it the third most dominant phylum. Chlorophyta was the predominant phylum in the winter (34.02%) and the second-dominant phylum in the spring (14.97%), but its abundance was low in the summer (2.49%) and autumn (1.92%). Ochrophyta was the second-dominant phylum in the summer (9.32%) and the third-dominant phylum in both the spring (8.25%) and the autumn (9.32%), with a lower relative abundance in the winter (4.54%) compared to other seasons. As the second-dominant phylum in both the autumn (14.92%) and the winter (8.35%), Cryptophyta gradually decreased from the autumn to the summer, where it accounted for only 0.52%. Conversely, Cryptomycota gradually increased in relative abundance from the autumn to the summer, eventually becoming the third-dominant phylum in the summer (5.06%).

Figure 4b shows the composition of the eukaryotic microbial communities based on the genus level. Excluding the unclassified genera, the dominant genera were identified including *Paracyclopina* (8.42%), *Sinocalanus* (5.46%), *Pseudodiaptomus* (2.41%), *Microcyclops* (1.47%), and *Limnocythere* (1.07%) belonging to Arthropoda; *Cryptomonas* (2.42%) and *Teleaulax* (1.64%) belonging to Cryptophyta; and *Discostella* (1.33%) belonging to Ochrophyta. *Paracyclopina* was the dominant genus in the spring (9.47%) and summer (19.59%), less abundant in the autumn (4.50%), and the least abundant in the winter (0.15%). *Sinocalanus* was the predominant genus in the autumn (7.27%) and the winter (4.93%), the second-dominant genus in the spring (7.72%), and had the lowest relative abundance in the summer (1.91%). Similarly, the lowest abundance of *Cryptomonas* occurred in the summer (0.10%), and higher proportions were in the spring (2.01%), autumn (5.19%), and winter (2.37%). *Teleaulax*, *Microcyclops,* and *Limnocythere* peaked in the autumn (5.55%, 5.74%, and 3.72%, respectively), while all of their relative abundance was less than 1% in the other seasons. *Discostella* was enriched in the spring (2.46%) and autumn (2.12%), but less frequent in the summer (0.27%) and winter (0.46%).

**Figure 5:**
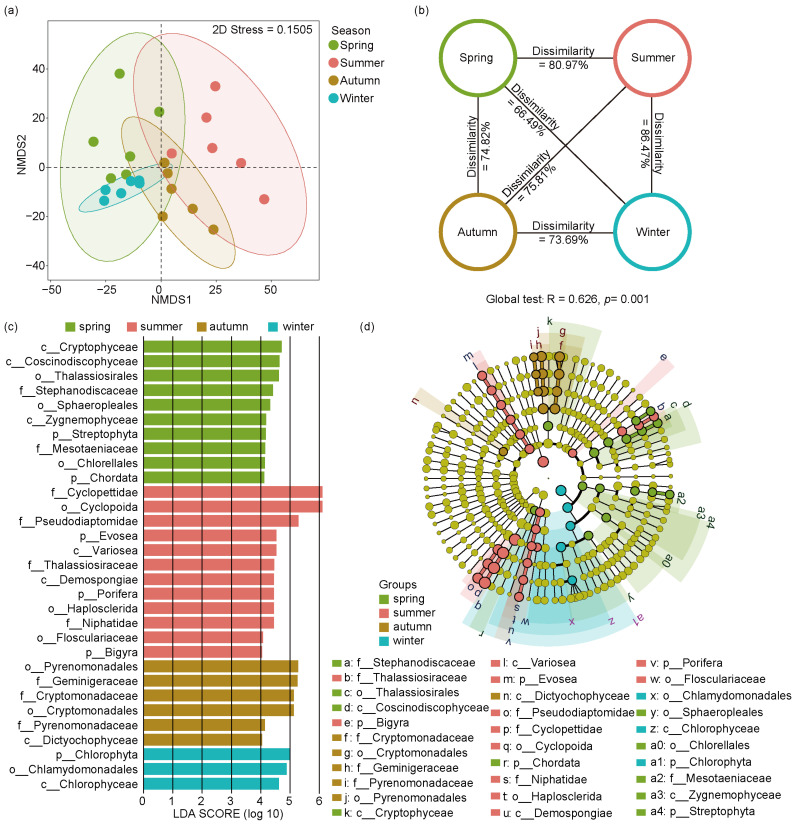
Non-metric multidimensional scaling analysis (NMDS), analysis of similarities (ANOSIM), and linear discriminant analysis effect size (LEfSe) analysis of eukaryotic microbial community within four seasons in Jihongtan Reservoir. (**a**) NMDS ordination plot produced based on Aitchison distance; (**b**) ANOSIM test; (**c**,**d**) LEfSe analysis; (**c**) Linear discriminant analysis (LDA) Score diagram shows differentially abundant taxa [LDA score = 4]; (**d**) Cladogram showing the phylogenetic structure of the eukaryotic microorganisms.

NMDS ordination and ANOSIM tests were used to analyze the seasonal differences of eukaryotic microbial communities across four seasons (Figure 5a,b). The NMDS results showed a distinct separation of seasonal changes in eukaryotic microbial communities (stress value = 0.15). In addition, the ANOSIM test of eukaryotic microbial communities showed that significant changes emerged in four seasonal groups (Global R = 0.626, *p* = 0.001), indicating population shifts of eukaryotic microorganisms among different seasons in the Jihongtan Reservoir.

To identify the eukaryotic biomarkers with a significantly different relative abundance across the four seasons, we utilized LEfSe (Figure 5c,d), choosing linear discriminant analysis scores greater than four to distinguish significantly different groups. Consequently, we identified 31 biomarkers throughout the year, with 10 in spring, 12 in summer, 6 in autumn, and 3 in winter. In spring, the identified biomarkers belonged mainly to Cryptophyta, Bacillariophyta, Chlorophyta, Streptophyta, and Chordata. In summer, the main enriched members belonged to Arthropoda, Evosea, Ochrophyta, Porifera, and Bigyra. In autumn, the biomarkers mainly included Dictyochophyceae and members from Cryptophyta. Chlorophyta and its subgroups, Chlorophyceae and Chlamydomonadales, were more prevalent in winter.

### 3.4. Correlations between Eukaryotic Microbial Communities and Environmental Variables

**Figure 6:**
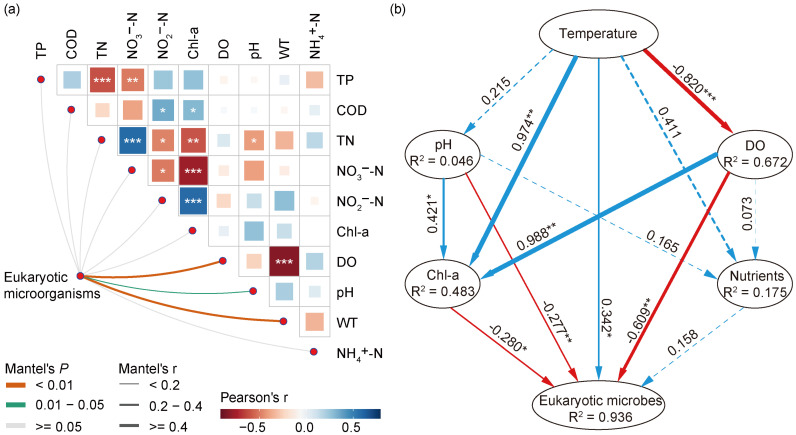
Environmental factors affecting eukaryotic microbial communities in Jihongtan Reservoir. (**a**) Pairwise comparisons of environmental factors are visually represented using a color gradient to indicate Spearman’s correlation coefficients. The correlations between the eukaryotic microbial community and each environmental factor are evaluated using Mantel tests. (**b**) Partial least squares path modeling (PLS-PM) represents the direct and indirect effects of environmental variables on eukaryotic microbial communities. The blue line: a positive relationship; the red line: a negative relationship. Significance level: *p* < 0.001 ***; *p* < 0.01 **; *p* < 0.05 *.

The shifts regarding the abundance of eukaryotic microbes at the phylum and genus levels, along with various environmental factors, indicated a strong link between the eukaryotic community and environmental changes. Thus, we conducted further research to better understand the relationship between eukaryotic microbial communities and environmental variables. The Mantel test (Figure 6a) revealed several important environmental variables, including WT (r = 0.441, *p* = 0.001), DO (r = 0.498, *p* = 0.001), and pH (r = 0.192, *p* = 0.033), that played a major role in the variabilities of eukaryotic microorganisms in different seasons, which showed a positive correlation with eukaryotic microbial communities (Table S1). Compared to pH, WT and DO showed a higher correlation. Furthermore, we observed correlations among various water quality parameters and found that DO was significantly negatively correlated with WT. 

How do the WT, DO, pH, and other environmental factors influence the eukaryotic microbial communities: through synergistic or direct ways? To address this issue, we constructed the partial least squares path model (PLS-PM), as shown in Figure 6b. The environmental drivers were categorized into six block variables: WT, pH, DO, nutrients (TN, TP, NH_4_^+^-N, NO_3_^−^-N, and NO_2_^−^-N), and algal properties (Chl-a). The result showed that the WT significantly influenced the eukaryotic microbial communities directly and positively, with the effects of 0.342 (*p* < 0.05), while DO, pH, and Chl-a showed a direct negative impact, with the effects of −0.609 (*p* < 0.01), −0.277 (*p* < 0.01), and −0.280 (*p* < 0.05), respectively. In addition, the WT exerted an indirect influence on the eukaryotic community by significantly affecting mainly DO and Chl-a, with the effects of −0.820 (*p* < 0.001) and 0.974 (*p* < 0.01), respectively. As a result, the WT could be the primary factor driving the seasonal succession of eukaryotic compositions in the Jihongtan Reservoir. 

### 3.5. Co-Existence Network Analysis

**Figure 7:**
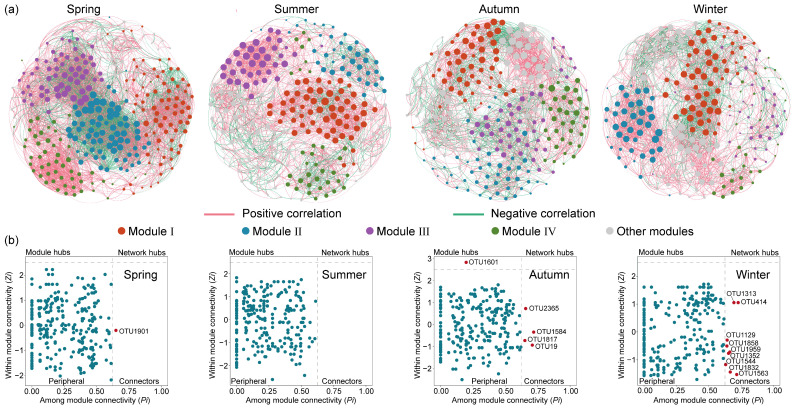
Seasonal co-occurrence network patterns of eukaryotic microbial communities in Jihongtan Reservoir. (**a**) Co-occurrence networks under different seasons; (**b**) keystone species analysis in different seasons.

Eukaryotic microbial communities are affected not only by abiotic factors, but also by biotic interactions. Co-occurrence networks of eukaryotic microbial communities under different seasons were constructed (Figure 7a) and the topological parameters are shown in Table S3. Across the four networks, the spring network exhibited a higher complexity, evident from its greater number of modes, linkage numbers, and highest average degree. Furthermore, the network in spring contained the highest graph density, shortest path length, and high clustering coefficient, indicating that spring eukaryotic microorganisms were more closely associated and correlated, with greater efficiency in the transferring of information, energy, and matter between taxa. Additionally, OTUs exhibited mainly positive correlations with each other, and this positive correlation is strongest in the summer and weakest in the autumn. In general, the positive interactions of the co-occurrence network represent cooperative or mutualistic relationships, while the negative correlations reflect competitive or antagonistic relationships [20]. The large proportion of positive links in the four seasons suggested that eukaryotic microbes are inclined to cooperate as a survival strategy in the Jihongtan Reservoir. 

Microbial networks can help identify keystone taxa, in addition to revealing co-occurrence patterns. According to the Zi (within-module connectivity) and Pi (among-module connectivity) values, nodes in a network can be categorized into four groups: network hubs (Zi > 2.5, Pi > 0.62), module hubs (Zi > 2.5, Pi < 0.62), connectors (Zi < 2.5, Pi > 0.62), and peripherals (Zi < 2.5, Pi < 0.62) [41]. Specifically, network hubs refer to nodes that can be highly connected within their own module as well as to other modules; module hubs are nodes that are only strongly connected within one module; connectors are nodes that connect different modules; and peripherals have limited connectivity within other nodes. In general, except for the peripherals, the other three categories are considered to contain keystone taxa due to their important roles in network topology, which may play a unique and crucial role in maintaining the microbiome structure and functioning [25]. According to Figure 7b, the majority of nodes in the four networks were peripheral, and only a minimal number of nodes were identified as key species. More detailed taxonomic information about keystones is presented in Table S2, indicating that the key species varied in both quantity and composition across the four seasonal communities. In the spring network, one connector was found, but as an unknown species. In the autumn network, one node assigned to the unknown species was identified as a module hub, and four nodes were identified as connectors belonging to Arthropoda, Porifera, and Cryptophyta. In the winter network, nine nodes were classified as connectors, of which four connectors were unassigned and the others mainly belonged to Ochrophyta, Ciliophora, and Chlorophyta. 

## 4. Discussion

### 4.1. Seasonal Dynamics of Eukaryotic Microbial Diversity and Structure

The analyses of OTUs and alpha diversity revealed that the eukaryotic microbial biodiversity was higher in the spring and summer compared to the autumn and winter. Fang et al. studied the microbiome characteristics of urban rivers in different seasons and the seasonal changes in diversity were consistent with our results, suggesting that such changes could be partly explained by temperature variations [42]. Typically, severe conditions, especially the lower temperatures, lead to low eukaryotic microbial diversity [37]. For example, a low temperature narrows the niche for species with different ecological requirements, resulting in a decline in the species number and a consequent decrease in the diversity [43]. Other things being equal, there are more species in warm environments than in cold ones [44]. Notably, we found that the microbial diversity in the Jihongtan reservoir showed no significant differences across the four seasons, which is inconsistent with the results of previous studies of reservoirs [17,45]. A water diversion is a possible reason that might explain this phenomenon. Exotic microorganisms along with various kinds of nutrients can be introduced into the reservoir through the water transfer project, contributing to altering microbial community diversity directly and indirectly [46,47]. Previous studies indicated that an external water transfer can impact the microbial diversity in the water-receiving area [48,49]. For instance, Yang et al. [50] discovered that the Shannon–Wiener diversity index, Margalef richness index, Simpson dominance index, and Pielou evenness index of phytoplankton in receiving rivers were increased greatly after the two water transfers in the summer and winter.

This study demonstrated that the microbial community structure exhibited significant seasonal changes. Arthropoda was the most dominant phylum in all seasons except winter. Most of the dominant genera belong to the phylum Arthropoda, including *Paracyclopina*, *Sinocalanus*, *Pseudodiaptomus*, and *Microcyclops*, all of which are assigned to Copepoda. The abundance of Copepoda is highest in the summer and lowest in the winter. As primary and secondary consumers, Copepoda transfers organic carbon and energy from primary producers to higher trophic levels, playing a major role in aquatic food webs [51]. The abundance of *Paracyclopina* was much higher than that of *Sinocalanus* in the summer, whereas in the winter, the opposite trend was observed, reflecting changes in the water eutrophication level to some extent [52]. Among the identified dominant phyla and biomarkers, a significant portion belonged to algae, which play an essential role in maintaining primary productivity and matter-energy flows as autochthonous autotrophic producers in aquatic ecosystems [7,53]. 

It is notable that Chlorophyta dominates in the spring and winter, whereas its relative abundance is low in the summer and autumn, which is not consistent with previous studies showing that the higher water temperature and abundant nutrient conditions are more suitable for the mass reproduction of Chlorophyta [17,54]. We speculate that water diversion is the possible reason that might explain this phenomenon. Some studies have found dramatic differences in the phytoplankton composition before and after water transfers in freshwater ecosystems [55,56]. Dai et al. [57] stated that the main causes of variations in lacustrine phytoplankton communities were the direct input of exogenous species and changes in aquatic habitat conditions of the receiving lake, rather than nutrient perturbation. It has been found that most of the Chlorophyta species have faster growth rates and are more adaptive to the turbulent flowing environment [58]. Yang et al. [50] observed that Chlorophyta in the urban river increased by a maximum increment of 150% after the water diversion project in winter. As illustrated in Table 1, the primary sources of water replenishment in the spring and winter are ESNWD and YQWD, and the water recharge amount for each season is approximately equal to the capacity of the Jihongtan Reservoir, of which, ESNWD provided a water recharge of 57% in the spring and 83% in the winter, indicating ESNWD may have a greater impact on the Jihongtan Reservoir in the spring and winter. Chlorophyta has also been found to be one of the dominant algae phyla in ESNWD [59,60]. Dongping Lake, as part of ESNWD, experienced a rapid increase in the proportion of Chlorophyta during the water transfer period in March [55]. Moreover, according to Figure 3, there were no significant changes in the relative abundance of Chlorophyta in the inlets and outlets. Taken together, we prefer that mainly direct inputs of exogenous microorganisms caused the high abundance of Chlorophyta in the spring and winter.

### 4.2. Environmental Factors and Eukaryotic Microbial Communities

In this study, the mantel test and PLS-PM were conducted to analyze the correlation between environmental variables and the eukaryotic community structure. Among the environmental factors measured in the reservoir, WT, pH, and DO were found to be significantly associated with the eukaryotic microbial community structure, of which WT was the most critical factor driving seasonal succession among eukaryotic microorganisms. Many studies have reported the key role of WT in regulating variation in eukaryotic microbial communities [29,61]. According to the results of PLS-PM analysis, we can explain the important role of WT in shaping community diversity from two aspects (direct and indirect impacts). On the one hand, the water temperature can directly impact the cellular activity and metabolic rates, thereby influencing the life cycle of microorganisms through their growth and metabolic capacity [62]. For instance, the Copedoda species shows a positive correlation with the water temperature [63], and favorable warmer conditions support the growth of copepods. On the other hand, the water temperature, as a key indicator of seasonal characteristics, could indirectly affect the eukaryotic community structure by changing other environmental variables, such as DO and Chl-a. Chl-a is widely used as an indicator of water column phytoplankton biomass within aquatic ecosystems [64], and Zhang et al. [17] reported that there are interactions between phytoplankton and micro-eukaryotes. Being a key factor for phytoplankton reproduction and metabolism, DO was significantly negatively correlated with WT, indicating that a higher water temperature contributed to the lower DO concentration and vice versa. Moreover, pH was observed to have an obviously negative impact on the community of eukaryotic microorganisms, and an elevated pH may affect the nutrient uptake by affecting enzyme synthesis and cellular osmotic pressure, consequently affecting growth, metabolism, and the other life activities of aquatic organisms [65,66]. 

### 4.3. Co-Occurrence Patterns of Eukaryotic Microbial Communities and Keystone Taxa

Co-occurrence networks could offer insight into microbial interactions, the network complexity and stability beyond just diversity and composition [67]. Some studies have found that molecular ecological networks of microbial communities exhibited clear seasonal patterns, and microbial stability distinctly varied with seasonal variations [42,68]. Water diversion projects have been found to directly impact the stability of the community structure, but a stable state was re-established with the continuation of long-term water diversion [59]. In our study, the robustness results show that the spring network (0.337 ± 0.023) demonstrated the highest robustness compared to the summer (0.295 ± 0.030), autumn (0.294 ± 0.030), and winter (0.287 ± 0.030), among which there is little difference in robustness, which was in accord with the results of the network complexity. This finding is in agreement with Liu et al. [27], who explored the variations of microbial stability among seasons in lake ecosystems and confirmed that networks with a higher complexity are more likely to be stable. This suggests that stronger interspecific interactions could enhance the stability of microbial communities, leading to better resistance to external environmental disturbances. Moreover, the highest modularity in the summer network, the strongest negative correlation in the autumn network, and the greatest number of key species in the winter network may contribute to the strength of stability [26,69]. Competitive relationships among microorganisms are critical for maintaining a stable ecological system. Although positive correlations within communities can promote mutual assistance among members, cooperation can create dependency and the potential for mutual downfall, leading to oscillations across the network when responding together to environmental changes. Negative connections can help stabilize co-oscillation within communities and contribute to the stability of networks as a high proportion of negative links could better balance the asynchronous dynamics [70,71]. Keystone taxa are important for maintaining microbial community stability, and their removal could lead to the breakdown of networks. Liu et al. [72] found that keystone taxa contributed a lot to maintaining the biological community stability in a seasonal shallow lake, strongly affected by changes in hydrological disturbances and nutrient inputs. The modular organization of species would be beneficial for the local stability of ecological communities by enabling them to recover from small disturbances [73]. Taken together, compared to modularity, negative correlations, and keystone taxa, we speculated that the complexity of the network played a more active and important role in eukaryotic microbial community network stability in the Jihongtan Reservoir in terms of year-round water diversion.

Keystone taxa were identified in the microbial community irrespective of their abundance across space and time, and key taxa were found to be rare species in some studies [18,27]. Recent research on the rare biosphere has been receiving increasing attention. Rare microbes exhibit high genetic diversity and consist of a large number of metabolically active lineages, playing fundamental roles in regulating the ecological function and biogeochemical processes of various aquatic systems [74,75]. In this study, keystone taxa consisted of twelve species with very low abundance rankings (<1%) and three species with high abundance rankings (>1%). In the autumn network, there were two of each rare and abundant species in the keystone taxa. However, in the spring and winter, with the highest water diversions, the key taxa were dominated by rare species. Li et al. posit that within low-stress environmental contexts, the abundant biosphere exerts a substantially more pronounced influence on the stabilization of ecological networks compared to the rare biosphere, but the difference between their relative importance was observed to diminish significantly with the increasing stress [76]. These results suggested that both abundant and rare species exert influence on maintaining eukaryotic microbial community stability in the Jihongtan Reservoir. Additionally, we found no occurrences of crossover species in the four season networks, which is consistent with the findings of previous studies [27,28], suggesting that keystone taxa, serving as the core hubs within a network, may exhibit environment specificity, being confined to particular seasons or time periods. 

## 5. Conclusions

This study investigated the seasonal characteristics of eukaryotic microbial communities in the Jihongtan Reservoir within year-round water diversion. The eukaryotic microbial diversity, community structure, and co-occurrence patterns showed seasonal variations, with significant changes observed in the community structure. The relationship between the microbial diversity and environmental factors was investigated, finding that the water temperature was the primary driver of seasonal succession in eukaryotic microbial communities, both directly and indirectly. The co-occurrence network and robustness results indicated that the complex eukaryotic microbial network showed more stability under the conditions of year-round water diversion. Keystone taxa of the eukaryotic microbes were identified, and it was found that both abundant and rare species play an important role in maintaining eukaryotic microbial community stability in the Jihongtan Reservoir. The impact of the water transfer project on the eukaryotic microbial community in the Jihongtan Reservoir is complex, due to constant variations in the water source and the amount of water diversion and, therefore, the continuous monitoring of aquatic organisms should be implemented. Our study results provide valuable insights for preserving the microbial diversity and aquatic ecosystem of the Jihongtan Reservoir in the future.

## Figures and Tables

**Table 1:** Cumulative inflow amount of water diversion project to Jihongtan Reservoir (10^8^ m^3^).

Sources	Spr	Sum	Aut	Win
ESNWD	0.7127	-	0.1607	1.2141
YQWD	0.5428	0.2630	-	0.2571
XR	-	0.2839	0.3845	-
Total	1.2555	0.5469	0.5452	1.4712

## Data Availability

The data presented in this study are openly available in NCBI (https://www.ncbi.nlm.nih.gov/, accessed on 18 August 2024), reference number PRJNA1137302.

## Supplementary Materials

The following supporting information can be downloaded at: https://www.mdpi.com/article/10.3390/microorganisms12091873/s1, Table S1: Mantel tests for the correlation between environmental variables and eukaryotic microbes based on Spearman rank correlation; Table S2: Topological parameters of eukaryotic microbial networks in four seasons; Table S3: Taxonomic information of keystone taxa in four season networks.
