# Supplementary material for: Seasonal Dynamics of Eukaryotic Microbial Communities in the Water-Receiving Reservoir of the Long-Distance Water Diversion Project, China
Source: Microorganisms. 2024 Sep 11;12(9):1873. doi: 10.3390/microorganisms12091873 (PMC11433762; doi:10.3390/microorganisms12091873)
Supplement: Supplementary file 1 [file microorganisms-12-01873-s001.zip › microorganisms-3178408-supplementary.pdf]

Table S1. Mantel tests for the correlation between environmental variables and eukaryotic microbes based on Spearman rank correlation.

Microbes	Environmental factors	r	p	Mantel's r	Mantel's p
Eukaryotic microbes	NH_4^+-N	-0.0928156	0.787	< 0.2	>= 0.05
	WT	0.44089363	0.001	>= 0.4	< 0.01
	pH	0.19159691	0.033	< 0.2	0.01 - 0.05
	DO	0.49799237	0.001	>= 0.4	< 0.01
	Chl-a	0.12932082	0.118	< 0.2	>= 0.05
	NO_2^--N	0.07319383	0.166	< 0.2	>= 0.05
	NO_3^--N	0.06073518	0.274	< 0.2	>= 0.05
	TN	0.03133556	0.357	< 0.2	>= 0.05
	COD	-0.0945152	0.863	< 0.2	>= 0.05
	TP	0.0457502	0.319	< 0.2	>= 0.05

Table S2. Topological parameters of eukaryotic microbial networks in four seasons.

Topological Parameters	Spr	Sum	Aut	Win
Number of Nodes	303	258	255	243
Number of Edges	5428	2630	2566	2634
Average Degree	35.828	20.388	20.125	21.679
Average weighted degree	31.944	18.150	17.859	19.204
Graph Density	0.119	0.079	0.079	0.090
Average Path Length	2.740	2.977	2.907	2.882
Average Clustering Coefficient	0.620	0.575	0.556	0.550
Modularity	0.543	0.633	0.605	0.532
Network diameter	7	6	6	6
Positive correlation (%)	66.56	74.26	62.20	68.15
Negative correlation (%)	33.44	25.74	37.80	31.85

Table S3. Taxonomic information of keystone taxa in four season networks.

Group	Role	OTUs	relative abundance	Phylum	Class	Genus
Spring	Module hubs	NA	NA	NA	NA	NA
	Network hubs	NA	NA	NA	NA	NA
	Connectors	OTU1901	0.04%	Unassigned	Unassigned	*Unassigned*
Summer	Module hubs	NA	NA	NA	NA	NA
	Network hubs	NA	NA	NA	NA	NA
	Connectors	NA	NA	NA	NA	NA
Autumn	Module hubs	OTU1601	0.34%	Unassigned	Unassigned	*Unassigned*
	Network hubs	NA	NA	NA	NA	NA
	Connectors	OTU19	4.50%	Arthropoda	Hexanauplia	*Paracyclopina*
		OTU1584	0.02%	Porifera	Demospongiae	*Ephydatia*
		OTU1817	0.27%	Cryptophyta	Cryptophyceae	*Cryptomonas*
		OTU2365	2.42%	Cryptophyta	Cryptophyceae	*Unassigned*
Winter	Module hubs	NA	NA	NA	NA	NA
	Network hubs	NA	NA	NA	NA	NA
	Connectors	OTU414	0.01%	Ochrophyta	Dictyochophyceae	*Unassigned*
		OTU1129	0.03%	Ciliophora	Spirotrichea	*Tintinnidium*
		OTU1313	0.01%	Unassigned	Unassigned	*Unassigned*
		OTU1352	1.01%	Chlorophyta	Chlorophyceae	*Chlamydomonas*
		OTU1544	0.01%	Unassigned	Unassigned	*Unassigned*
		OTU1563	0.07%	Ochrophyta	Fragilariophyceae	*Fragilaria*
		OTU1832	0.16%	Unassigned	Unassigned	*Unassigned*
		OTU1858	0.40%	Unassigned	Unassigned	*Unassigned*
		OTU1959	0.03%	Unassigned	Unassigned	*Unassigned*

NA: Not Available
